# Bidirectional Ventricular Tachycardia with Stress-Induced Cardiomyopathy

**DOI:** 10.1155/2022/1065847

**Published:** 2022-04-27

**Authors:** Ariyon Schreiber, Michael Gardner, Chris Sossou, Natasha Greene, Chowdhury Ahsan

**Affiliations:** ^1^University of Nevada Las Vegas School of Medicine, Department of Medicine, USA; ^2^Department of Cardiology, University of Nevada, Las Vegas, Las Vegas, USA

## Abstract

Bidirectional ventricular tachycardia (BDVT) is a rare electrocardiographic finding characterized by rapid, wide complex, alternating QRS morphology with 180-degree swings in the frontal plane axis or, less commonly, alternating right bundle branch and left bundle branch block morphology. The most proposed mechanisms for BDVT involve triggered activity or enhanced automaticity resulting from calcium dysregulation. Catecholamine surge can cause myocardial injury as well as calcium dysregulation resulting in enhanced automaticity that can lead to arrhythmias such as BDVT. This case report stands to describe a unique presentation of BDVT and stress-induced cardiomyopathy, resulting from catecholamine surge following multiple traumatic gunshot wounds in the setting of methamphetamine use.

## 1. Introduction

Bidirectional ventricular tachycardia (BDVT) is a unique phenomenon that has fascinated electrophysiologists since its first description in 1922 [1]. Characterization of BDVT on electrocardiogram (ECG) most commonly includes rapid, wide complex, and alternating QRS morphology with 180-degree swings in the frontal plane axis, and less commonly includes alternations of right bundle branch and left bundle branch forms [2]. Various etiologies for BDVT have been proposed, including digitalis toxicity, hypokalemia, Andersen-Tawil syndrome, acute myocarditis, and catecholaminergic polymorphic ventricular tachycardia (CPVT). Most of the proposed mechanisms involve triggered activity or enhanced automaticity resulting from calcium dysregulation [2]. We describe a unique case of BDVT, resulting from a catecholamine surge following multiple traumatic injuries in the setting of methamphetamine use. Furthermore, the BDVT described in this case was associated with a stress-induced cardiomyopathy, suggestive of Takotsubo's cardiomyopathy, which resolved within days.

## 2. Case Report

A 22-year-old male with no significant past medical history, including no history of arrhythmia or syncope, presented with multiple gunshot wounds to the face and bilateral upper extremities. The patient was intubated en route for airway protection.

**Figure 1:**
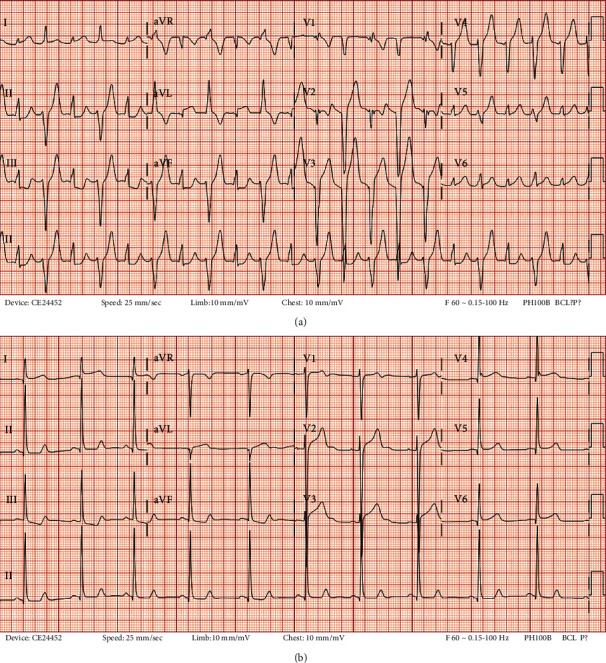
(a) ECG of patient upon presentation to the ED, demonstrating characteristics of BDVT. (b) 4 hours later: repeat ECG found to be normal.

Upon arrival to trauma resuscitation, the patient was noted to be tachycardic at 112-113 beats per minute, hypertensive with a systolic pressure > 200 mmHg (peak 241 mmHg), and saturating O_2_ at 100%. Initial ECG demonstrated characteristics consistent with BDVT, including tachycardia with a left-axis deviation pattern alternating with a right-axis deviation pattern, as well as an alternating bundle branch block (Figure 1(a)). Repeat ECGs were found to be normal at both twenty minutes and four hours (Figure 1(b)).

Patient's labs were significant for mild hypokalemia, minimally elevated hs-troponin-I, and significant leukocytosis (Table 1). Urine drug screen demonstrated recent use of methamphetamine, benzodiazepines, and tetrahydrocannabinol.

**Figure 2:**
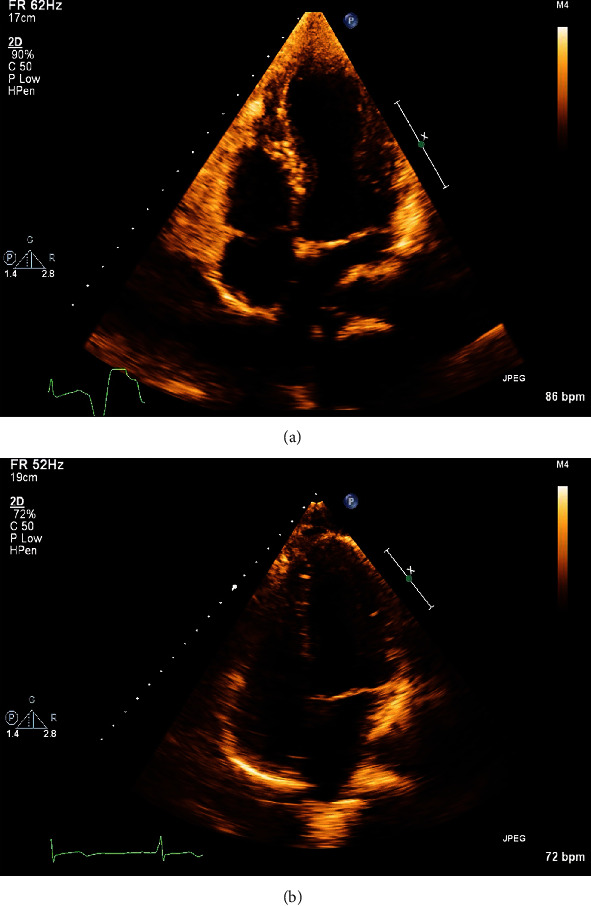
(a) TTE apical 4-chamber view in systole, demonstrating apical ballooning, suggestive of Takotsubo's stress-induced cardiomyopathy. (b) 14 days later: TTE apical 4-chamber view in systole, demonstrating resolution of patient's Takotsubo's stress-induced cardiomyopathy.

Transthoracic echocardiography (TTE) demonstrated midleft ventricular (LV) apical hypokinesis and apical ballooning, basal LV hyperkinesis, and mildly reduced left ventricular systolic function (LVSF) consistent with stress-induced cardiomyopathy (Figure 2(a)). Recorded values included an end-diastolic volume (EDV) of 59.3 mL, an end-systolic volume (ESV) of 26.8 mL, and an ejection fraction (EF) of <55%.

Following numerous surgical interventions, the patient was successfully extubated and discharged on aggressive guideline-directed medical therapy (GDMT), per 2017 recommendations from the Journal of the American College of Cardiology (JACC) for mortality reduction in patients with reduced ejection fraction (EF) [3], including carvedilol 12.5 mg twice daily, lisinopril 5 mg once daily, and spironolactone 25 mg once daily. The patient was also instructed to follow up on an outpatient basis with cardiology. Due to patient's age and suspicion of prompt recovery, a repeat echo was performed 14 days later. Compared to the initial echocardiogram, LVSF improved to normal function, including increases in EDV to 109.6 mL, ESV to 40.6 mL, and EF to >55%. The left ventricle was visualized to be normal in size and without regional wall motion abnormalities (Figure 2(b)).

## 3. Discussion

BDVT is defined as a tachycardia showing beat-to-beat alternations in the QRS axis of ventricular complexes in at least some of the ECG leads [4]. Similar to cases reported in the literature, our ECG displays

1. a left-axis deviation pattern alternating with a right-axis deviation pattern and visualization of an alternating bundle branch block [4]
2. tachycardia occurring in a short burst, lasting only a few minutes, and resolving spontaneously
3. wide QRS complexes [4]
4. a regular rhythm despite an underlying rhythm of atrial fibrillation [4]

The exact etiology of our patient's BDVT is ultimately unknown. Though digitalis toxicity is the most common cause of BDVT, our patient reported no cardiac history and denied past or present digoxin use. Further, our patient was not taking any known herbal supplementation, eliminating concern for aconite poisoning. He had no past history nor familial history of cardiac arrest, ryanodine receptor mutations (RYR2), and/or neurologic deficits, ruling out Andersen-Tawil syndrome and decreasing the likelihood of CPVT, though this differential cannot be completely eliminated due to the potential of sporadic RYR2 mutations and lack of genetic exploration and counseling. Finally, he had no signs nor symptoms of recent viral illness, myocardial infarction, valvular disease, left ventricular hypertrophy, cardiac sarcoidosis, ischemic cardiomyopathy, or acute coronary syndrome, ultimately winnowing out a number of alternative etiologies. This patient's unique presentation of BDVT prompted exploration of the literature to best describe the mechanism and etiology involved.

Multiple mechanisms have been proposed for BDVT. One of the most prominent proposed mechanisms includes dysregulation of intracellular calcium leading to elevated levels that cause delayed afterdepolarization in anatomically separate parts of the conducting system. Baher et al. [5] proposed that two separate foci, with different rate thresholds for delayed afterdepolarization-induced ventricular bigeminy, were present in a rabbit model [6]. When the ventricular rate exceeded the lower threshold, bigeminy would develop. This would effectively double the heart rate, increasing the overall ventricular rate above the second threshold. Once this has developed, the two competing sites would simply alternate on a beat-to-beat basis. This is likely the mechanism underlying BDVT observed with digitalis toxicity and CPVT.

Genetic investigators have demonstrated that most families with CPVT have mutations in genes regulating the level and/or function of proteins responsible for the balance of intracellular myocyte calcium [7]. In addition to autosomal dominant mutations in the ryanodine receptor (RYR2), mutations in calsequestrin-2 (CasQ2), Kir2.1-inward-rectifier potassium channel (KCNJ2), calmodulin (CALM1), and triadin (TRDN) have been identified in families with the clinical picture of CPVT [7]. Most models to explain the disease focus on RyR2 mutations, as they account for more than half of diagnosed cases and multiple mutations have been identified [7]. The mouse models of RyR2 mutations suggested that delayed afterdepolarizations are triggered in all myocytes when exposed to known stressors, such as caffeine and adrenaline, or spontaneously [6].

It is likely in our case that in the setting of multiple traumas and recent methamphetamine use, severe dysregulation of intracardiac calcium channels from catecholamine toxicity led to the manifestation of this unusual and rare arrhythmia. It is well known that methamphetamine use can lead to catecholamine toxicity by binding vesicular monoamine transporter 2, accumulating vesicles of dopamine in the synaptic cleft and norepinephrine in the cytosol, inhibiting enzyme monoamine oxidase to stabilize cytosolic catecholamine levels, inhibiting dopamine and norepinephrine transporters preventing catecholamine reuptake, and stimulating catecholamine release through exchange diffusion and modulation of receptor activity. This results in an acute upsurge in dopamine and norepinephrine, resulting in catecholamine toxicity and consequential calcium dysregulation.

Catecholamine toxicity explains not only the rare manifestation of BDVT but also our patient's stress-induced cardiomyopathy. It has been suggested that catecholamine surge can cause damage to cardiomyocytes by two primary mechanisms. The first mechanism is characterized by calcium overload, consecutive beta-adrenergic receptor-mediated activation of protein kinase A, and subsequent phosphorylation of multiple Ca^2+^-cycling proteins, including sarcolemmal L-type Ca^2+^ channels, phospholamban, and sarcoplasmic reticulum ryanodine receptor Ca^2+^ release channels (RyR2) [7]. Increased intracellular calcium and calcium dysregulation lead to increased automaticity of the cardiac myocyte, resulting in this momentary manifestation of BDVT. The second mechanism is characterized by oxidative stress, primarily related to the transformation of catecholamines into “aminochromes.” Aminochromes then undergo redox cycling in mitochondria to generate copious amounts of oxygen-derived free radicals [8]. This oxidative damage may have led to our patient's stress-induced cardiomyopathy and later reversal. Initiating aggressive GDMT likely played a role in this patient's spontaneous recovery. These two mechanisms can explain both our patient's manifested BDVT and his stress-induced cardiomyopathy.

With regard to the diagnosis of Takotsubo's cardiomyopathy, recent studies performed using the international Takotsubo registry have found a scoring system that can accurately differentiate Takotsubo syndrome from acute coronary syndrome without the use of coronary angiography with 89% sensitivity and 91% specificity [9]. Patients with scores of greater than 50 confer a 95% specificity for Takotsubo cardiomyopathy [9]. Our patient was found to have an underlying psychiatric disorder, absence of ST-segment changes on ECG, and physical and emotional triggers conferring a score of 61. Coronary angiography was deferred in his case due to several emergent surgical interventions, numerous comorbidities, and low suspicion for acute coronary syndrome. The patient was instructed to follow up outpatient with cardiology for angiography once stable; unfortunately, his records indicate that he has been, thus far, lost to follow up.

In conclusion, there is a great deal of data to explain the connection between BDVT, stress-induced cardiomyopathy, and catecholamine surge; however, there are no systematic studies to demonstrate evidence of connection. Herein, we provide the first-ever documented case of BDVT manifested alongside a stress-induced cardiomyopathy with apical morphology triggered by catecholamine surge.

## Figures and Tables

**Table 1:** Important laboratory values.

Laboratories	Reference	Day 1	Day 2	Day 3	Day 4	Day 5
White blood cell	3.10-10.20 K/mm^3^	17.16 (H)	18.38 (H)	15.03 (H)	13.57 (H)	12.87 (H)
Hemoglobin	13.1-16.8 g/dL	8.1 (L)	7.4 (L)	7.1 (L)	9.6 (L)	9.9 (L)
Platelets	119-332 K/mm^3^	173	179	169	240	275
Magnesium	1.60-2.60 mg/dL	1.80	1.72	1.74	1.61	1.61
Sodium	136-145 mmol/L	139	137	138	135	136
Potassium	3.5-5.1 mmol/L	3.2 (L)	4.5	4.0	4.6	4.1
Creatinine	0.55-1.3 mg/dL	0.91	0.86	0.86	0.72	.79
Calcium	8.4-10.2 mg/dL	7.7 (L)	8.3 (L)	8.3 (L)	7.2 (L)	8.2
Troponin I	0.020-0.040 ng/mL	10.669 (H) 9.168 (H) 7.467 (H)	6.224 (H) 3.798 (H) 2.072 (H)	1.753 (H) 1.807 (H)	—	—
pH, arterial	7.350-7.450	7.404	7.385	7.440	—	—
pCO_2_, arterial	35.0-45.0 mmHg	40.5	47.1 (H)	37.6	—	—
pO_2_, arterial	80.0-100 mmHg	465 (H)	148 (H)	190 (H)	—	—
O_2_ Sat, arterial	90.0-100.0%	>100.5 (>)	99.9	100.0 (H)	—	—
PEEP	cmH_2_O	5	5	—	—	—
FIO_2_	%	100.0	40.0	46.0	—	—

## Data Availability

All data is available within this case report.
